# Evapotranspiration Estimation with Small UAVs in Precision Agriculture

**DOI:** 10.3390/s20226427

**Published:** 2020-11-10

**Authors:** Haoyu Niu, Derek Hollenbeck, Tiebiao Zhao, Dong Wang, YangQuan Chen

**Affiliations:** 1 Electrical Engineering and Computer Science Department, University of California, Merced, CA 95340, USA; hniu2@ucmerced.edu; 2 Mechanical Engineering Department, University of California, Merced, CA 95340, USA; dhollenbeck@ucmerced.edu (D.H.); tzhao3@ucmerced.edu (T.Z.); 3 USDA-ARS, San Joaquin Valley Agricultural Sciences Center, Parlier, CA 93648, USA; Dong.Wang@usda.gov

**Keywords:** clumped canopy, evapotranspiration, unmanned aerial vehicles, METRIC, remote sensing

## Abstract

Estimating evapotranspiration (ET) has been one of the most critical research areas in agriculture because of water scarcity, the growing population, and climate change. The accurate estimation and mapping of ET are necessary for crop water management. Traditionally, researchers use water balance, soil moisture, weighing lysimeters, or an energy balance approach, such as Bowen ratio or eddy covariance towers to estimate ET. However, these ET methods are point-specific or area-weighted measurements and cannot be extended to a large scale. With the advent of satellite technology, remote sensing images became able to provide spatially distributed measurements. However, the spatial resolution of multispectral satellite images is in the range of meters, tens of meters, or hundreds of meters, which is often not enough for crops with clumped canopy structures, such as trees and vines. Unmanned aerial vehicles (UAVs) can mitigate these spatial and temporal limitations. Lightweight cameras and sensors can be mounted on the UAVs and take high-resolution images. Unlike satellite imagery, the spatial resolution of the UAV images can be at the centimeter-level. UAVs can also fly on-demand, which provides high temporal imagery. In this study, the authors examined different UAV-based approaches of ET estimation at first. Models and algorithms, such as mapping evapotranspiration at high resolution with internalized calibration (METRIC), the two-source energy balance (TSEB) model, and machine learning (ML) are analyzed and discussed herein. Second, challenges and opportunities for UAVs in ET estimation are also discussed, such as uncooled thermal camera calibration, UAV image collection, and image processing. Then, the authors share views on ET estimation with UAVs for future research and draw conclusive remarks.

## 1. Introduction

Evapotranspiration (ET) estimation is important for precision agriculture, especially precision water management. Mapping the ET temporally and spatially can identify variations in the field, which is useful for evaluating soil moisture [1,2] and assessing crop water status [3]. ET estimation can also benefit water resource management and weather forecast [4]. ET is a combination of two separate processes, evaporation (E) and transpiration (T). Evaporation is the process whereby liquid water is converted to water vapor through latent heat exchange [5]. Transpiration is the process of the vaporization of liquid water contained in plant tissues, and the vapor removal to the atmosphere [5]. The current theory for transpiration is constituted by the following three steps. First, the conversion of liquid-phase water to vapor water causes canopy cooling from latent heat exchange. Thus, canopy temperature can be used as an indicator of ET. Second, diffusion of water vapor from inside plant stomata on the leaves to the surrounding atmosphere. Third, atmospheric air mixing by convection or diffusion transports vapor near the plant surfaces to the upper atmosphere or off-site away from the plant canopy. Usually, evaporation and transpiration occur simultaneously.

Many approaches have been developed to estimate ET. Typically, there are direct and indirect methods. For direct methods, ET can be determined by water balance [6]:

(1) ET=P+I−D−R−S,

where *P* (mm day${}^{-1}$) is precipitation, *I* (mm day${}^{-1}$) is irrigation, *D* (mm day${}^{-1}$) is drainage, *R* (mm day${}^{-1}$) is runoff, and S (mm day${}^{-1}$) is the soil moisture storage. These direct ET methods, however, are usually point-specific or area-weighted measurements and cannot be extended to a large scale because of the heterogeneity of the land surface. The experimental equipment is also costly and requires substantial expense and effort, such as lysimeters, which are only available for a small group of researchers. For indirect methods, there are energy balance methods [7] and remote sensing methods [8]. For energy balance methods, Bowen ratio [9,10] and eddy covariance [11] have been widely used in ET estimation. However, they are also area-weighted measurements. Remote sensing techniques can detect variations in vegetation and soil conditions over space and time. Thus, they have been considered as some of the most powerful methods for mapping and estimating spatial ET over the past decades [12,13]. Remote sensing models have been useful in accounting for the spatial variability of ET at regional scales when using satellite platforms such as Landsat and ASTER [14,15,16,17]. Since the satellite started being applied [18], several remote sensing models have been developed to estimate ET, such as surface energy balance algorithm for land (SEBAL) [8,15], mapping evapotranspiration with internalized calibration (METRIC) [19], the dual temperature difference (DTD) [20], and the Priestley–Taylor TSEB (TSEB-PT) [21]. Remote sensing techniques can provide information such as normalized difference vegetation index (NDVI), leaf area index (LAI), surface temperature, and surface albedo. Related research on these parameters has been discussed by different researchers [22,23,24].

As a new remote sensing platform, researchers are very interested in the potential of small UAVs for precision agriculture [25,26,27,28], especially on heterogenous crops, such as vineyard and orchards [29,30]. UAVs overcome some of the remote sensing limitations faced by satellite. For example, satellite remote sensing is prone to cloud cover; UAVs are below the clouds. Unlike satellites, UAVs can be operated at any time if the weather is within operating limitations. The satellite has a fixed flight path; UAVs are more mobile and adaptive for site selection. Mounted on the UAVs, lightweight sensors, such as RGB cameras, multispectral cameras, and thermal infrared cameras, can be used to collect high-resolution images. The higher temporal and spatial resolution images, relatively low operational costs, and the nearly real-time image acquisition, make the UAVs an ideal platform for mapping and monitoring ET. Many researchers have already used UAVs for ET estimation, as shown in Table 1. For example, in [31], Ortega-Farías et al. implemented a remote sensing energy balance (RSEB) algorithm for estimating energy components in an olive orchard, such as incoming solar radiation, sensible heat flux, soil heat flux, and latent heat flux. Optical sensors were mounted on a UAV to provide high spatial resolution images. By using the UAV platform, experiment results show that the RSEB algorithm can estimate latent heat flux and sensible heat flux with errors of 7% and 5%, respectively. It demonstrated that UAV could be used as an excellent platform to evaluate the spatial variability of ET in the olive orchard.

There are two objectives for this paper. First, to examine current applications of UAVs for ET estimation. Second, to explore the current uses and limitations of UAVs, such as UAVs’ technical and regulatory restrictions, camera calibrations, and data processing issues. There are many other ET estimation methods, such as surface energy balance index (SEBI) [41], crop water stress index (CWSI) [42], simplified surface energy balance index (S-SEBI) [43], and surface energy balance system (SEBS) [12], which have not been applied with UAVs. Therefore, they are out of the scope of this article. This study is not intended to provide an exhaustive review of all direct or indirect methods that have been developed for ET estimation.

The rest of the paper is organized as follows: Section 2 introduces different UAV types being used for ET estimation. Several commonly used lightweight sensors are also compared in Section 2. The ET estimation methods being used with UAV platforms, as shown in Table 1, are discussed. In Section 3, different results of ET estimation methods and models are compared and discussed. Challenges and opportunities, such as thermal camera calibration, UAV path planning, and image processing, are discussed in Section 4. Lastly, the authors share views regarding ET estimation with UAVs in future research and draw conclusive remarks.

## 2. Materials and Methods

### 2.1. Unmanned Aerial Vehicles (UAVs) and LightWeight Sensors

**Figure 1:**
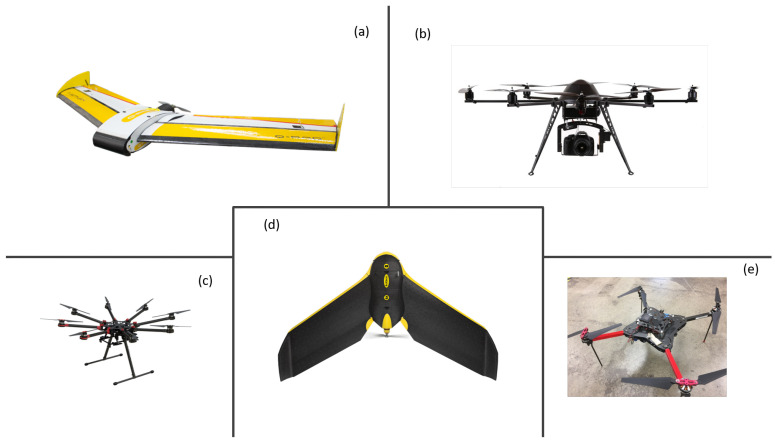
(**a**) The QuestUAV 200 UAV. (**b**) The MK Okto XL 6S12. (**c**) The DJI S1000. (**d**) The eBee Classic. (**e**) The Hover.

Many kinds of UAVs are used for different research purposes, including ET estimation. Some popular UAV platforms are shown in Figure 1. Typically, there are two types of UAV platforms, fixed-wings and multirotors. Fixed-wings can usually fly longer with a larger payload. They can usually fly for about 2 h, which is suitable for a large field. Multirotors can fly about 30 min, which is suitable for short flight missions. Both of them have been used in agricultural research, such as [30,44], which promises great potential in ET estimation.

Mounted on UAVs, many sensors (Table 2) can be used for collecting UAV imagery, such as multispectral and thermal images, for ET estimation. For example, the Survey 3 (MAPIR, San Diego, CA, USA) camera has four bands, blue, green, red, and near-infrared (NIR), with a spectral resolution of 4608 × 3456 pixels, and a spatial resolution of 1.01 cm/pixel. (Mention of trade names or commercial products in this publication is solely for the purpose of providing specific information and does not imply recommendation or endorsement by the University of California or the U.S. Department of Agriculture. The University of California and the USDA are equal opportunity providers and employers.) The Survey 3 camera has a fast interval timer, 2 s for JPG mode, and 3 s for RAW + JPG mode. Faster interval timer would benefit the overlap design for UAV flight missions, such as reducing the flight time, and enabling higher overlapping. Another multispectral camera being commonly used is the Rededge M. The Rededge M has five bands, which are blue, green, red, near-infrared, and red edge. It has a spectral resolution of 1280 × 960 pixel, with a 46${}^{\circ }$ field of view. With a Downwelling Light Sensor (DLS), which is a 5-band light sensor that connects to the camera, the Rededge M can measure the ambient light during a flight mission for each of the five bands. Then, it can record the light information in the metadata of the images captured by the camera. After the camera calibration, the information detected by the DLS can be used to correct lighting changes during a flight, such as changes in cloud cover during a UAV flight.

The thermal camera ICI 9640 P (Infrared Cameras Inc, Beaumont, TX, USA) has been used for collecting thermal images as reported in [45,46,47,48]. The thermal camera has a resolution of 640 × 480 pixels. The spectral band is from 7 to 14 μm. The dimensions of the thermal camera are 34 × 30 × 34 mm. The accuracy is designed to be ±2 ${}^{\circ }$C. A Raspberry Pi Model B computer (Raspberry Pi Foundation, Cambridge, UK) can be used to trigger the thermal camera during flight missions. The SWIR 640 P-Series (Infrared Cameras Inc., Beaumont, TX, USA), which is a shortwave infrared camera, can also be used for ET estimation. The spectral band is from 0.9 μm to 1.7 μm. The accuracy for the SWIR camera is ±1 ${}^{\circ }$C. It has a resolution of 640 × 512 pixels.

### 2.2. ET Estimation Methods with UAVs

Most ET estimation using UAVs is based on satellite remote sensing methods. One source energy balance (OSEB), high resolution mapping of evapotranspiration (HRMET) [49], machine learning (ML) [50,51,52,53,54,55], artificial neural networks (ANN) [56], two source energy balance (TSEB), dual-temperature-difference (DTD) [57], the surface energy balance algorithm for Land (SEBAL) [8,15], and mapping evapotranspiration at high resolution with internalized calibration (METRIC) [19] are introduced in this section. The discussed ET estimation methods with UAVs and their advantages and disadvantages are summarized in Table 3. As mentioned earlier, this article is not intended to provide an exhaustive review of all direct or indirect methods that have been developed for ET estimation, but rather to provide an overview on ET estimation with UAVs applications. Therefore, only those methods (Table 1) which have already been used with the UAVs platform are discussed.

#### 2.2.1. One Source Energy Balance (OSEB)

One source energy balance (OSEB) model assumes the whole surface as a uniform layer. OSEB model does not differentiate potential sources, such as the soil and canopy [12,15]. The OSEB model uses empirical parameters to explain differences in the aerodynamic and radiometric components [58,59,60,61,62]. The OSEB model uses the following equation to calculate the latent heat flux,

(2) $$LE=R_{n}-G-H,$$

where LE is the latent heat flux (W m${}^{-2}$), $R_{n}$ is the net radiation (W m${}^{-2}$), *G* is the soil heat flux (W m${}^{-2}$), and *H* is the sensible heat flux (W m${}^{-2}$). The sensible heat flux *H* is calculated by

(3) $$H=\rho C_{p}\frac{T_{aero}-T_{ac}}{R_{ah}},$$

where ρ is the air density (kg m${}^{-3}$), $C_{p}$ is the specific heat of air (J kg${}^{-1}$ K${}^{-1}$), $T_{aero}$ is the aerodynamic temperature (K) [63], and $T_{ac}$ is the air temperature (K) in the vegetation [64,65,66]. $R_{ah}$ is the aerodynamic resistance to heat flux (s m${}^{-1}$), which is calculated by

(4) $$R_{ah}=\frac{\left[\ln\left(\frac{z_{u}-d}{z_{om}}\right)-\Psi_{m}\right]\left[\ln\left(\frac{z_{t}-d}{z_{om}}\right)+\ln\left(\frac{z_{om}}{z_{oh}}\right)-\Psi_{h}\right]}{k^{2}u},$$

where $z_{u}$ and $z_{t}$ are the wind and air temperature measurement heights, respectively. The parameter d is the zero displacement height, $z_{om}$ is the momentum transfer [67,68], $\Psi_{m}$ and $\Psi_{h}$ are the diabatic correction factors for momentum and heat [69], $z_{oh}$ is the resistance to heat, *k* is the Karman constant, and *u* is the wind speed.

The parameter $kB^{-1}$ is also used in OSEB model to adjust the radiometric to the aerodynamic temperature. The measured radiometric temperature can be used in Equation (4) instead of $T_{aero}$. The parameter $kB^{-1}$ is calculated by

(5) $$kB^{-1}=\ln\left(\frac{z_{om}}{z_{oh}}\right).$$

There are also some other types of OSEB models. For example, deriving atmosphere turbulent transport useful to dummies using temperature (DATTUTDUT) [70] is an energy balance model which only needs the land surface temperature as the input for ET estimation. The DATTUTDUT estimates ET by scaling the energy fluxes between the hot and cold pixels. The DATTUTDUT model is similar to the simplified surface energy balance index (S-SEBI) proposed by Roerink [43]. However, the DATTUTDUT model is more simplified to acquire the radiometric temperature. More details can be found in [70].

#### 2.2.2. High Resolution Mapping of Evapotranspiration (HRMET)

Most current ET models, such as METRIC and SEBAL, are highly reliant on selecting hot and cold pixels to separate energy fluxes between latent and sensible heat in the images. Therefore, their ability is limited to map ET throughout the growing season at extremely high spatial resolutions. Thus, Zipper et al. [49] developed a field-validated surface energy balance model, which is called high resolution mapping of evapotranspiration (HRMET). The HRMET only requires basic meteorological data, spatial surface temperature, and canopy structure data. For more detailed calculation steps of the HRMET, please refer to [49].

#### 2.2.3. Machine Learning (ML) and Artificial Neural Networks (ANN)

Machine learning techniques and ANN models have already been used for estimating hydrological parameters [50,51,52,53,54,55] and ecological variables [71]. Due to the ML’s ability to capture non-linear characteristics, many research results suggest that machine learning methods can provide better ET estimates than empirical equations based on different meteorological parameters [72,73,74,75,76,77,78,79]. Traditional multispectral indices have limitations with assessing water status. Therefore, artificial neural networks (ANN) were used in [56] to improve the estimation of spatial variability of vine water status. In [80], Dou et al. used four different machine learning approaches in different terrestrial ecosystems for ET estimation. ANN, support vector machine (SVM), extreme learning machine (ELM) [81], and adaptive neuro-fuzzy inference system (ANFIS) [78,82,83,84,85,86] were compared with each other on estimating ET. In [87], Torres-Rua et al. built a narrowband and broadband emissivities model for UAV thermal imagery using a deep learning (DL) model. The resulting emissivities were incorporated into the TSEB model to analyze their effects on the estimation of instantaneous energy balance components against ground measurements.

#### 2.2.4. Two Source Energy Balance (TSEB) Models

The TSEB model is developed to improve the accuracy of LE estimation [20,24,64,88], using the assumptions of canopy transpiration in Priestley and Taylor potential evapotranspiration [89] calculations. Therefore, this TSEB model is also called TSEB-PT to differentiate it from other TSEB methods. The calculations of sensible heat flux and latent heat flux for canopy and soil are separate, which makes the parameterization of resistances easier compared with a single layer model. Based on [90,91], the TSEB is effective over homogeneous land and environmental conditions. The TSEB model reproduces fluxes with similar results to tower-based observations.

The TSEB model separates the land surface temperature into soil surface temperature $T_{s}$ and vegetation surface temperature $T_{c}$. Subscripts “s” and “c” mean soil and canopy. It considers that sensible and latent heat fluxes are transferred to the atmosphere from both surface temperature components, as shown in the following equations [35].

(6) $$R_{n}=R_{ns}+R_{nc},$$

(7) $$R_{ns}=H_{s}+LE_{s}+G,$$

(8) $$R_{nc}=H_{c}+LE_{c}.$$

The net radiation $R_{n}$ is divided into two parts, the soil net radiation $R_{ns}$ and the canopy net radiation $R_{nc}$ [92,93].

(9) $$R_{ns}=\tau_{l}L_{d}+\left(1-\tau_{l}\right)\varepsilon_{c}\sigma T_{c}^{4}-\varepsilon_{s}\sigma T_{s}^{4}+\tau_{s}\left(1-\alpha_{s}\right)S_{d},$$

(10) $$R_{nc}=\left(1-\tau_{l}\right)\left(L_{d}+\varepsilon_{s}\sigma T_{s}^{4}-2\varepsilon_{c}\sigma T_{c}^{4}\right)+\left(1-\tau_{s}\right)\left(1-\alpha_{c}\right)S_{d},$$

where $\tau_{l}$ and $\tau_{s}$ are the longwave and shortwave radiation transmittances through the canopy, respectively. $L_{d}$ and $S_{d}$ are the incoming longwave and shortwave radiation (W m${}^{-2}$), which are usually measured from a nearby weather station. The Stefan–Boltzmann constant is given by σ, which is approximately 5.67 × 10${}^{-8}$ (W m${}^{-2}$ K${}^{-4}$). The surface emissivity is denoted by ε, α is the surface albedo, and *T* is the surface temperature (K).

For the soil sensible heat flux $H_{s}$ and canopy sensible heat flux $H_{c}$, they can be calculated based on the following equations,

(11) $$H_{s}=\rho C_{p}\frac{T_{s}-T_{ac}}{R_{s}},$$

(12) $$H_{c}=\rho C_{p}\frac{T_{c}-T_{ac}}{R_{x}},$$

where ρ is the air density (kg m${}^{-3}$), $C_{p}$ is the specific heat of air (J kg${}^{-1}$ K${}^{-1}$), $T_{ac}$ is the air temperature in the vegetation [64,65,66], $R_{s}$ is the resistance to heat flux above the soil surface (s m${}^{-1}$), and $R_{x}$ is the boundary layer resistance of the canopy leaves (s m${}^{-1}$).

#### 2.2.5. Dual-Temperature-Difference (DTD) Model

The DTD model separates the land surface temperature into vegetation and soil temperatures [57]. Then, it calculates the surface energy balance components by using the same procedures as the TSEB. The TSEB model is very sensitive to the temperature difference between the land surface and air, which makes the absolute land surface temperature inaccurate. To solve this problem, the DTD model added one more input dataset, the land surface temperature retrieved one hour after sunrise. The energy fluxes are minimal at sunrise, which minimizes the bias in the temperature estimation. For the soil sensible heat flux $H_{s}$ and canopy sensible heat flux $H_{c}$, Equations (11) and (12) become

(13) $$H_{s}=\rho C_{p}\frac{\left(T_{s_{i}}-T_{s_{o}}\right)-\left(T_{ac_{i}}-T_{ac_{o}}\right)}{R_{s}},$$

(14) $$H_{c}=\rho C_{p}\frac{\left(T_{c_{i}}-T_{c_{o}}\right)-\left(T_{ac_{i}}-T_{ac_{o}}\right)}{R_{x}},$$

where subscript *i* means the measurements are at midday and subscript *o* refers to observations one hour after the sunrise.

In [94], Guzinski et al. produced surface energy flux successfully by using the DTD model with satellite images; they used night observations to substitute for the early morning observation. However, the temporal resolution of the satellite observations is limited, especially when the weather conditions are limiting. For example, satellite thermal infrared observations cannot penetrate clouds when there is cloud cover. The incapacity to collect data in overcast situations applies to all satellite sensors except for those operating in the microwave region [94].

The calculation of soil heat flux *G* is different between midday and sunrise observations. This difference can be used to estimate the soil surface temperature variations. Soil heat flux is calculated based on the model of [95]. The soil heat flux equation is

(15) $$G=R_{ns}Acos\left(2\pi \frac{\left(t+10800\right)}{B}\right),$$

(16) $$A=0.0074\Delta T_{R}+0.088,$$

(17) $$B=1729\Delta T_{R}+65013,$$

where $\Delta T_{R}$ is the diurnal variation in the soil surface temperature, and t is the time between the data collection time and the solar noon. For more details about the TSEB and DTD equations, see [96,97].

#### 2.2.6. Surface Energy Balance Algorithm for Land (SEBAL)

The surface energy balance algorithm for land (SEBAL) model uses surface temperature $T_{s}$, visible, near-infrared, thermal infrared radiation, albedo maps, and NDVI to estimate surface fluxes with many different land cover types [15,98]. SEBAL is currently one of the most reliable algorithms with which to estimate actual ET ($ET_{a}$), and it is one of the most promising approaches currently for local and regional estimation with minimum ground data [7]. SEBAL has been validated in many different climatic conditions around the world [99,100,101,102,103]. Typically, the SEBAL model’s accuracy is around 85% and 95% at daily and seasonal scales, respectively [99,104]. To calculate ET as a residual of the energy balance model, the sensible heat flux *H* needs to be estimated first.

In the SEBAL model, two reference air temperatures are measured to compute the air temperature difference (dT). One air temperature is measured at the height $h_{1}$ close to the surface. The other is measured at an upper height of $h_{2}$. To calculate dT for each pixel, SEBAL assumes that there is a linear relationship between dT and the surface temperature $T_{s}$ as

(18) $$dT=a+bT_{s},$$

where *a* and *b* are derived parameters empirically based on two extreme hot and cold pixels, also called “anchor” pixels [98]. These hot and cold pixels defined the boundary of the sensible heat flux. The cold pixel is usually selected from a well-watered area with no water stress. The *H* is assumed to be minimum, and ET should be maximum. The hot pixel is taken from a dry and bare field where *H* is maximum, and ET is almost zero. Hot and cold pixels are usually selected by an empirical method.

Most SEBAL applications for estimating energy fluxes and ET have used spaceborne platforms with a relatively low spatial resolution. There is not much-published work related to the use of the SEBAL model to estimate ET using UAVs. Selecting hot or cold pixels is subjective, which can cause variations in ET estimation. Estimated sensible heat flux *H* is easily affected by the surface-air temperature differences or surface temperature measurements. The radiometer’s viewing angle can also cause variations in $T_{s}$ by several degrees for some images.

Although SEBAL has limitations, there are also several advantages of SEBAL for estimating land surface fluxes from thermal remote sensing data. First, SEBAL does not need a lot of ground-based data. Second, SEBAL has automatic internal correction. Third, every image has an internal calibration in SEBAL.

#### 2.2.7. Mapping Evapotranspiration at High Resolution with Internalized Calibration (METRIC)

METRIC is originally a satellite image processing model for estimating ET as a residual of the energy balance [19], which is based on SEBAL. The METRIC can generate ET maps with a 30-meter spatial resolution by using Landsat images. METRIC has a self-calibration process which contains ground-based hourly reference ET and the selection of hot, cold pixels [105]. It eliminates the need for absolute surface temperature calibration [8].

SEBAL uses $T_{s}$, ρ, NDVI, and their relationships to calculate the surface fluxes [15], which has been evaluated all over the world [99,100,101,102,103]. The METRIC model uses the same method as the SEBAL model to estimate dT. Thus, there is no need to get an accurate aerodynamic surface temperature. In [7], Liou et al. summarized three differences between the SEBAL and METRIC. First, for the cold pixel, the METRIC does not consider sensible heat flux as zero. Instead, a surface soil water balance is applied to set ET as 1.05 times reference ET at cold pixels. Reference ET is calculated by using the standardized American Society of Civil Engineers (ASCE) Penman–Monteith equation. Second, in METRIC, cold pixels are selected in agricultural settings instead of biophysical characteristics. Third, the extrapolation of instantaneous ET is based on reference ET instead of the actual evaporative fraction.

METRIC estimates ET using the energy balance Equation (2). For the net radiation $R_{n}$ (W m${}^{-2}$), it can be calculated by adding all the incoming radiation and subtracting all the outgoing radiation based on the following equation [19].

(19) $$R_{n}=\left(1-\alpha \right)R_{s↓}+R_{L↓}-R_{L↑}-\left(1-\varepsilon_{o}\right)R_{L↓},$$

where $R_{s↓}$ is the incoming short-wave radiation (W m${}^{-2}$); α is the surface albedo; $R_{L↓}$ and $R_{L↑}$ are the incoming longwave radiation (W m${}^{-2}$) and outgoing long-wave radiation (W m${}^{-2}$), respectively. $\varepsilon_{o}$ is the thermal emissivity, which is also dimensionless. These parameters can be calculated in METRIC with several submodels that use other parameters derived from the ground-based weather data, digital elevation model (DEM), and satellite images [19].

Sensible heat flux *H* (W m${}^{-2}$) is computed from surface roughness, wind speed, surface temperature ranges,

(20) $$H=\rho_{air}C_{p}\frac{dT}{r_{ah}},$$

where $r_{ah}$ is the aerodynamic resistance (s m${}^{-1}$) between two surface height. In METRIC, $r_{ah}$ is usually calculated by using the wind speed, LAI or NDVI, and an iterative stability correction, as shown in the following equation,

(21) $$r_{ah}=\frac{\ln\left(z_{2}/z_{1}\right)}{u_{*}k},$$

where $z_{1}$ and $z_{2}$ are heights above the zero-plane displacement of the vegetation. *k* is the von Karman constant (0.41). $u_{*}$ is the friction velocity (m s${}^{-1}$), which is calculated by using

(22) $$u_{*}=\frac{ku_{200}}{\ln\left(200/z_{om}\right)},$$

where $u_{200}$ is the wind speed at a blending height 200 m, and $z_{om}$ is the momentum roughness length (m).

The temperature difference between the air and the surface is represented by dT. A strong linear relationship between the dT and the surface temperature was found in [15,19,104,106], as shown in Equation (18). The sensible heat fluxes for the cold and hot pixels are calculated by Equation (2). According to [107]; for the cold pixel, the ratio LE and $ET_{r}$ is assumed to be 1.05. However, this assumption is not always true at the beginning or outside of the growing season when the vegetation is much less than the alfalfa [18]. Therefore, the ratios of the LE and $ET_{r}$ for the cold and hot pixels are calculated by NDVI [19]. Then, the dT and land surface temperature $T_{s}$ for the cold and hot pixels are applied for calculating the *a* and *b* in Equation (18) as

(23) $$a=\frac{dT_{hot}-dT_{cold}}{T_{s_{hot}}-T_{s_{cold}}},$$

(24) $$b=\frac{dT_{hot}-a}{T_{s_{hot}}},$$

where $T_{s_{hot}}$ and $T_{s_{cold}}$ are the land surface temperature (K) at the hot and cold pixels, respectively.

## 3. Results and Discussion

Compared with traditional satellite remote sensing approaches, the UAVs platform and the lightweight cameras can estimate the surface energy fluxes with similar accuracy. Therefore, the UAVs can be used for modeling ET estimation with high confidence. In this section, different crops’ ET estimations with UAV platforms (Table 4) are compared with each other. The reasons behind the errors of ET estimation are also discussed in related sections.

### 3.1. OSEB and TSEB Models

In [40], Brenner et al. compared the OSEB model with the TSEB model by using an octocopter MikroKopter OktoXL (HiSystems GmbH, Moormerland, Germany). This UAV platform can carry a payload of 4 kg for each flight mission. An ES80 camera ( Samsung, Seoul, South Korea) and an Optris Pi 400 thermal camera were mounted on the UAV to collect images. According to the specification, Pi 400 had an accuracy of ±2 ${}^{\circ }$C. The thermal image resolution was 382 × 288 pixels with a field of view 38${}^{\circ }$× 29${}^{\circ }$. Approximately 700 to 1000 thermal images were collected for every flight mission. The eddy covariance system was used to evaluate the UAV ET estimation.

Based on the comparison between UAV fluxes and eddy covariance (EC) fluxes, the net radiation $R_{n}$ for TSEB is in good agreement with $R_{n}$ measured from EC with an R-squared value ($R^{2}$) of 0.99. The $R^{2}$ value for OSEB is 0.98. The sensible heat flux (*H*) for TSEB has a $R^{2}$ value of 0.84, and the OSEB has a $R^{2}$ value of 0.79. For the soil heat flux *G*, the $R^{2}$ value for OSEB is 0.73. The TSEB has a $R^{2}$ value of 0.83. Both models underestimated the ground heat flux compared with the eddy covariance system. For the latent heat flux LE, OSEB has a $R^{2}$ value of 0.92. The TSEB has a $R^{2}$ value of 0.93.

**Remark** **1.** *The results show that the OSEB model significantly underestimated measured values for flux conditions. The poor performance of the OSEB model mainly results from an underestimation of high fluxes. Differently from the TSEB model, the OSEB model needs an empirical adjustment parameter $kB_{-1}$ to explain the difference between the radiometric and aerodynamic surface temperature. The parameter $kB_{-1}$ is usually overestimated in case of strong temperature gradients between the surface and the atmosphere [40].*

### 3.2. HARMET Model

In [16], Park et al. used the HARMET model when flying a DJI S1000 UAV. A thermal infrared camera A65 and a multispectral camera Rededge M were mounted on the UAV to collect thermal and multispectral images. The thermal camera image resolution is 640 × 512 pixels with a field of view of 25${}^{\circ }$× 20${}^{\circ }$. The Rededge had a spatial resolution of 1280 × 960 pixels. The UAV was flown at solar noon for capturing the period of high ET and for minimizing tree canopy shadows.

The energy fluxes were estimated in the HRMET model. For the reference trees, the estimated ET was around 0.62 mm h${}^{-1}$. The study site was small and the UAV flight time was less than 15 min; thus, the meteorological data, such as incoming shortwave radiation, wind speed, and vapor pressure, were considered to be consistent during the UAV flight mission. The different ET rates along the trees were mainly decided by the differences in tree canopy temperature and LAI. The estimated ET had a strong linear relationship with leaf transpiration (R${}^{2}$ = 0.9).

**Remark** **2.** *Although it was challenging to evaluate the results because of the absence of sufficient data, such as the directly measured ET or multi-seasonal UAV data, the HARMET model still showed great potential for estimating tree-by-tree ET and capturing intra-field variability.*

### 3.3. Machine Learning and Neural Networks

In [56], Poblete et al. used ANN and multispectral images from a UAV platform to predict vine water status. A multispectral camera MCA-6 (Tetracam Inc, Chatsworth, CA, USA) was mounted on an octocopter Mikrokopter OktoXL for data collection. The data were grouped into training and validation, where 80% was used for the ANN model calibration, and 20% was used to validate the model. Although this research was not exactly for ET estimation, it proved that Neural Networks, such as ANN, had a great potential for ET estimation when combing with high-resolution multispectral UAV images.

In [80], four machine learning methods, ANN, SVM, ELM, and ANFIS, were used to estimate ET. Results showed that all four models could detect the variations of ET. The reason is that ML algorithms can identify complex non-linear relationships between ET and environmental variables. As a new model, ELM exhibits strong modeling accuracy for daily ET estimation. ANFIS can estimate ET more efficiently than ANN and SVM. More importantly, these new machine learning approaches show a novel perspective for ET estimation with remote sensing data. Therefore, UAV platforms should be used with ML algorithms together, which will have great potential for ET estimation in the future.

### 3.4. TSEB and DTD Models

The UAVs can help generate more accurate maps of NDVI, LAI, $f_{c}\left(\theta \right)$, and $T_{R}\left(\theta \right)$, which are the critical input data for the TSEB and DTD models [108]. In [21], Hoffmann et al. used the TSEB model and the DTD model when flying a Q300, which has a 2.2 m wingspan and can carry a payload of 2 kg for a 25-minute flight. An Optris PI 450 camera was mounted on the UAV to collect thermal images. Hoffmann et al. concatenated the LST thermal images into the orthomosaic, which were applied as the input for TSEB model [21]. According to the specifications, the thermal camera has an accuracy of ±2 ${}^{\circ }$C or ±2% at an ambient temperature of 23 ± 5 ${}^{\circ }$C. The thermal image resolution is 382 × 288 pixels at 90 m flying height. Around 700 to 1000 thermal images were collected for every flight mission. The eddy covariance system was used for a comparison with the UAV results.

Based on the comparison between UAV fluxes and eddy covariance (EC) fluxes, the net radiation $R_{n}$ for TSEB is in good agreement with $R_{n}$ measured from EC with a root mean square error (RMSE) of 44 W m${}^{-2}$ (11%); the correlation coefficient was 0.98. The sensible heat flux (*H*) for DTD had a RMSE of 59 W m${}^{-2}$ (64%), and the mean absolute error (MAE) value was 49 W m${}^{-2}$ (52%). Compared with DTD, the TSEB model had a RMSE of 85 W m${}^{-2}$ (91%) and the MAE was 75 W m${}^{-2}$ (81%). The TSEB had a better linear relationship between measured sensible heat flux *H* and modeled *H*. The soil heat fluxes (*G*) were underestimated—RMSE and MAE of 48 W m${}^{-2}$ (91%) and 45 W m${}^{-2}$ (86%) for DTD, respectively. The RSME and MAE for TSEB were 38 W m${}^{-2}$ (72%) and 35 W m${}^{-2}$ (66%), respectively. The correlation between the modeled *G* and measured *G* was very poor. Soil heat flux *G* is measured with the heat flux plates, which can lead to uncertainties in measured *G* [109]. For the latent heat flux LE, DTD had RMSE and MAE of 67 W m${}^{-2}$ (26%) and 57 W m${}^{-2}$ (22%), respectively. The TSEB has RMSE and MAE values of 94 W m${}^{-2}$ (37%) and 84 W m${}^{-2}$ (33%), respectively.

**Remark** **3.** *The results show that the DTD model predicted the energy fluxes better than TSEB, which demonstrates that by adding another input, the land surface temperature was retrieved one hour after sunrise, make the DTD model more robust. It concluded that the thermal camera placed on a UAV platform could provide high spatial and temporal resolution data for estimating energy balance fluxes of ET. Calibration of the thermal camera is also likely to improve TSEB heat flux computations. This study shows similar results as Guzinski’s work [96], who applied the TSEB at the same site but using satellite images instead of UAV images. In [96], the RMSE is 46 W m${}^{-2}$ for $R_{n}$, 56 W m${}^{-2}$ for H, and 66 W m${}^{-2}$ for LE. The DTD model in [21] achieved RMSE of 44 W m${}^{-2}$ for $R_{n}$, 59 W m${}^{-2}$ for H and 67 W m${}^{-2}$ for LE.*

### 3.5. TSEB and DATTUTDUT Models

Xia et al. [35] used the TSEB model and DATTUTDUT model for a sub-field and plant canopy scale ET monitoring over vineyards. Based on the results, the TSEB model estimated sensible heat flux and latent heat flux with the RMSE ranging from 20 to 60 W m${}^{-2}$. DATTUTDUT estimated heat fluxes with a larger error; the RMSE for latent heat flux LE was 105 W m${}^{-2}$. The net radiation $R_{n}$ had an RMSE of 65 W m${}^{-2}$. It concluded that the TSEB model could simulate the energy balance components in two vineyards with MAE ranging from 15 to 90 W m${}^{-2}$. They found that the TSEB model was fairly robust and was able to calculate LE and ET values under varying environmental conditions. By using the TSEB, the $T_{s}$ and $T_{c}$ had a bias of 0.5 ${}^{\circ }$C and RMSE in the order of 2.5 ${}^{\circ }$C. The accuracy was similar to [64,65,88,110], in which the RMSE values were between 2.4 and 5.0 ${}^{\circ }$C for $T_{s}$ and 0.83 and 6.4 ${}^{\circ }$C for $T_{c}$.

**Remark** **4.** *In general, the TSEB has a better performance than the DATTUTDUT model. The reason might be that the TSEB has a better physical representation of the energy exchange. DATTUTDUT, as a single-source model, is more sensitive when the surface temperature pixels are selected [111,112]. The actual extremes may not even exist in the thermal images. Besides, the effect of aerodynamic resistance (surface roughness) is also not considered in the DATTUTDUT model.*

Ortega et al. [31] used the TSEB model to estimate the energy balance fluxes over a drip-irrigated olive orchard by using a helicopter-based UAV platform. The UAV flight height was at 60 m, which enabled the thermal camera’s image at 6 cm spatial resolution. For the multispectral camera Mini MCA-6, the resolution was 3.3 cm. The remote sensing energy balance (RSEB) algorithm was well implemented, and only the climatic parameters, such as air temperature $T_{a}$ and wind speed *u*, were measured as the input data. The UAV images were used for calculating the NDVI and soil temperature $T_{s}$. Ortega et al. used the Bowen ratio approach to balance $\left(R_{n}-G\right)$ and (H+LE) to close the energy balance.

For the net radiation $R_{n}$, the RMSE and MAE were 38 W m${}^{-2}$ and 33 W m${}^{-2}$, respectively. For the estimated soil heat flux *G* by TSEB, the RMSE and MAE were 19 W m${}^{-2}$ and 16 W m${}^{-2}$, respectively. Results showed that the algorithm estimates LE and *H* with errors of 7% and 5%, respectively. The RMSE and MAE for the sensible heat flux *H* were 56 W m${}^{-2}$ and 46 W m${}^{-2}$, respectively. The RMSE and MAE for latent heat flux LE were 50 W m${}^{-2}$ and 43 W m${}^{-2}$, respectively. That shows that the largest differences for *H* and LE were found when the wind speed was greater than 2.7 m s${}^{-1}$.

**Remark** **5.** *The results indicated that the UAV could be an excellent tool to evaluate the effects of spatial variability for ET estimation. The high spatial resolution images were able to show significant differences between the energy balance fluxes above the tree canopy and the soil surface. It concluded that the TSEB model was fairly robust and could estimate ET at a sub-field scale level under different environmental conditions. UAV can also help the satellite platforms for estimating intra-field spatial variability of the energy fluxes to improve the estimation of water requirements of sparse canopies, for example, orchards and vineyards, which have different plant densities and fractional covers.*

### 3.6. SEBAL Model

In [32], Montibeller et al. used the SEBAL model to estimate energy fluxes and ET of corn and soybean in Ames, Iowa. The UAV being used was the eBee Ag (SenseFly, Cheseaux-sur-Lausanne, Switzerland), which weighed about 700 g and could cover a 12 km${}^{2}$ area in one flight. A modified S110 camera (Canon Inc, Ota City, Tokyo, Japan), the Sequoia multispectral sensor (MicaSense, Seattle, WA, USA), and the thermoMAP camera (SenseFly, Cheseaux-sur-Lausanne, Switzerland) were equipped with the UAV to collect data for running the SEBAL model. The thermal and multispectral images are the most important data for this project. UAV images were collected from different growing stages of the crops, such as flowering, yield formation, and the ripening. The seasonal variability of ET and energy fluxes were also considered. Surface albedo and surface reflectance were measured by a spectroradiometer.

To evaluate the accuracy of estimated energy fluxes, [32] used linear regression models and residual plots methods. All pixels in the energy flux images were averaged to be compared with the observed values measured from the flux towers. The R${}^{2}$ for the net radiation $R_{n}$ predicted by SEBAL was 0.71, which was underestimated by about 17% compared with the flux towers. Underestimation was most likely caused by clouds at the time when UAV was flying. The net radiation $R_{n}$ ranged from 427.24 to 688.76 W m${}^{-2}$ during the UAV flight missions, with a RMSE of 6.09 W m${}^{-2}$.

Estimating soil heat flux *G* is the most challenging part. The estimated soil heat flux was compared with two soil heat plates in the test field. For the soil heat flux *G*, the R${}^{2}$ for the plate 1 is 0.17, with the RMSE of 11.23 W m${}^{-2}$. The R${}^{2}$ for the plate 2 is 0.22, with the RMSE of 31.02 W m${}^{-2}$. Both show a poor correlation. There are two main reasons behind it. First, the accuracy of the soil heat flux plates is very low. The grown canopy can cover the soil surface, which gives errors for soil heat flux estimations. The soil heat flux plates can detect the heat rate flow difference when the canopy is developing during the growing season. Second, the flux plates’ depth and the soil types also affect the heat flux estimation [109]. The soil heat flux G ranged from 14.57 to 119.76 W m${}^{-2}$ for the whole growing season, which was not a good estimation. Several factors could affect the soil heat flux values, such as the quality of the UAV images, the spatial distribution of surface albedo. The SEBAL model estimates G as the function of surface albedo, vegetation index, and surface temperature, which depends on the empirical equation developed by [98]. This equation was originally developed for Mediterranean regions; thus, different climatic conditions may have different results.

For the sensible heat flux, it requires an internal calibration method. The challenge is how to select hot and cold pixels appropriately. To solve this challenge, Montibeller et al. [32] created a water body for the cold pixel selection by placing an evaporative pan. The evaporative pan, however, differs from a natural water body, which affects the calculation of net radiation $R_{n}$ and soil heat flux *G*. Therefore, the anchor pixels are usually selected from the coldest pixels in the UAV images. The R${}^{2}$ for the sensible heat flux *H* is 0.5, with the RMSE of 8.84 W m${}^{-2}$. The estimated value by SEBAL overestimated the sensible heat flux by 5%. The sensible heat flux within the field was around 91.84 W m${}^{-2}$ during the growing season.

The R${}^{2}$ of the latent heat flux LE was 0.82, with an RMSE of 2.67 W m${}^{-2}$. The research result shows that the LE varies as the crop grows. The ET rate is also relevant to the crop growth stage. Corn, for example, has higher ET rates up until the tassel appears. The maximum mean for LE is 564.90 W m${}^{-2}$, and the minimum mean is 256.22 W m${}^{-2}$.

The relationship between NDVI and ET was also evaluated by the author while using SEBAL. It assumes that there is a linear relationship between NDVI and ET. However, the correlation between the NDVI and ET is very poor; the R${}^{2}$ is around 0.01. One of the reasons is that soil wetting may affect NDVI prediction [14]. Further study needs to be explored.

**Remark** **6.** *Overall, the research proves that the SEBAL model can be used for estimating ET with UAVs. The MAE and RMSE values show that SEBAL can estimate ET with the UAV images very well. UAVs platform also has great potential to help farmers making decisions with real-time crop conditions in the near future, which can monitor the water consumption of each crop in the field. The SEBAL algorithms being used by [32] were automated by reprogramming the model with Python, which improved the data processing for ET estimation.*

### 3.7. METRIC and METRIC-HR Models

METRIC is discussed here because of its potential in UAV applications. For satellite images, monthly images can be effective for estimating seasonal ET [19] by the METRIC model. However, during times of rapid vegetative growth, multiple dates of satellite images may be needed, which are usually not available because of limitations of the satellite revisit cycles. UAVs have more flexible flight schedules. Since METRIC is designed to use satellite images as inputs, several adjustments are usually needed for the high-resolution UAVs’ input data [32].

In [33], a modified METRIC model called METRIC high resolution (METRIC-HR) was proposed to use the UAVs high-resolution images. There are several differences between the METRIC and METRIC-HR. First, the digital elevation model (DEM) has a higher image resolution in METRIC-HR. Manal replaced the original DEM with a 15 cm resolution DEM, which was generated by using the Photoscan (Agisoft, St. Petersburg, Russia). Second, the National Land Cover Database (NLCD) is also replaced by a 15 cm NLCD in METRIC-HR, which can be used to develop NLCD high-resolution maps. Third, METRIC uses shortwave infrared (SWIR) bands generated by Landsat 8. The SWIR is usually used for calculating the normalized difference water index. SWIR value is usually less than zero for water, which can help identify water more accurately than NDVI. In METRIC-HR, SWIR was neglected because there was no water in the study site. The thermal band (TIR) resampling of METRIC-HR is also different from the METRIC model. The thermal band resolution being used in METRIC-HR is acquired by AggieAir, which has a 60 cm resolution. Since METRIC requires all bands to have identical resolutions, TIR resampling is necessary. Nearest neighbor resampling was performed in ArcGIS software, which did not change the original pixel values [113,114]. The shortwave radiance images (BGR) also have higher reflectance than Landsat 8 images. Therefore, upscaling BGR with Landsat 8 PSF and developing correction equations are necessary for the METRIC-HR model.

As mentioned earlier in the METRIC model section, selecting hot and cold pixels as anchor pixels can be subjective and requires experience. Different hot and cold pixels can lead to significant deviations in the final ET estimation [115]. METRIC recommends selecting cold pixels in a homogenous, well-watered place where the range of NDVI is from 0.76 to 0.84. The surface albedo range is from 0.18 to 0.24. Hot pixels are selected in a homogeneous bare, dry soil location with NDVI less than 0.2. The surface albedo for hot pixels is recommended to be from 0.17 to 0.23. More information about anchor pixels selection can be found in [19,116]. After METRIC and METRIC-HR models were run, the final output was the instantaneous $ET_{r}F$ (fraction of the alfalfa-based reference ET). For the METRIC-HR results, the $ET_{r}F$ values ranged from 0 to 1.15. Lower values represented hotter areas, such as bare soil; higher values meant wet areas. Compared with METRIC, METRIC-HR had a higher $ET_{r}F$ estimated; this could be caused by the presence of pixels of multiple vegetation growth with significant differences in some covers. The maximum difference was around 20%.

**Remark** **7.** *The results showed the values estimated between METRIC and METRIC-HR had a 0.9 coefficient of correlation. This proves that METRIC-HR has a similar performance to METRIC. Higher resolution images in the METRIC-HR model has a better performance in mixed areas. This work demonstrates that UAVs equipped with lightweight cameras can estimate ET quantitatively. However, cameras need further calibration to relate spectral response to METRIC-HR models.*

## 4. Challenges and Opportunities

Compared with traditional remote sensing tools, such as satellites, a UAV’s flight can be more flexible and frequent in the field. UAVs can fly at a lower altitude and can take higher spatial and temporal resolution images [46] of crops. As a low-cost scientific data collection platform, UAVs also make data acquisition relatively less expensive. While there are many advantages of using UAVs for ET estimation, there are still challenges for UAVs when used for estimating ET. These challenges are also commonly shown in the reviewed research work [16,21,31,32,33,34,35,36,37,38,39,40].

### 4.1. UAVs

Although UAVs have shown great potential for ET estimation, there are still many technical problems for UAVs, such as flight time, flight height control, path planning, stability in wind, and turbulence [117,118]. For example, most UAVs can only fly around 30 min with a payload, which is not enough for a large field. The power can also run low faster because of unexpected headwinds or other factors. Increasing the payload of UAVs will make the UAVs more capable. Flight height is another concern; in the United States, the maximum altitude for UAVs is limited to 120 m. The UAVs need to be in sight of the operator, and a pilot’s license is also required. Consequently, it is necessary to have a flying team for UAVs. For a detailed discussion on technical limitations for UAVs, please refer to [119]. Fortunately, it is expected that with the development of UAV technology, new camera designs, lower costs, improved image processing techniques, and a greater number of experimental studies of UAV-based remote sensing for agriculture applications, UAVs will have better performance for ET estimation.

### 4.2. UAV Path Planning and Image Processing

Many researchers fly the UAVs at different heights, using specialized equipment, controlled environments, and reliance on data analysis expertise [120]. Is there any optimal point where the data can be the best representation of a crops ET? In [120], Stark et al. built a conceptual framework for describing the optimality as a function of spatial, spectral, and temporal factors that represent the best solution. As researchers try to understand the potential of the UAVs, efficient workflow, image processing methods, and better software are still under development [121,122,123,124].

#### 4.2.1. Pre-Flight Path Planning

Being used for ET estimation as a remote sensing platform, UAVs also create new research problems, such as UAV image processing and flight path planning. Flight missions are usually designed by different kinds of software. The flight height is usually set up as 30, 60, or 120 m. For all the flight missions in the reviewed papers, the overlap was usually set up between 75% to 85% to enable the images to be stitched together during image processing. The UAV’s sensors are designed to take images at nearly 0 nadir angle.

Researchers usually fly UAVs biweekly to collect data. If there is a UAV crash, unexpected weather conditions, hardware issues, or unknown reasons, data may not be collected successfully. If data is missed, people may have to wait for another year. A bi-weekly UAV flight schedule is suggested to collect sufficient data.

#### 4.2.2. Multispectral Image Calibration

To minimize the shading effect on the images, the UAVs are usually flown at noon with clear sky conditions. As each pixel in a UAV image is a percentage of the reflected light, pixel values need to be calibrated by using a known reflectance value. Therefore, the image of a calibration board needs to be taken before and after the flight missions, thereby serving as the reflectance reference.

It is important to take pictures of the reference panel immediately before and after the flight missions because the solar angle and light intensity can change [125], which causes inaccurate experiment results. The digital number of the images are converted to reflectance by [126]

(25) $$\rho_{\lambda }=\frac{DN-DN_{d}}{DN_{w}-DN_{d}},$$

where $\rho_{\lambda }$ is the reflectance and DN is the digital number of the raw image; $DN_{d}$ and $DN_{w}$ are the dark reflectance point and white reflectance point in the color checker, respectively.

UAV images usually have higher radiometric homogeneity than aircraft or satellite images because of the lower flight altitude [127]. However, there are also special UAV image quality problems. For example, the camera position on the UAV might be different for each flight mission, which can cause different spatial resolution or different viewing angles [127]. The low flight height of UAVs can also result in geometric distortion [127,128]. Besides, lower flight height results in greater numbers of UAV images to keep effective overlapping, which makes image processing more time-consuming.

Although multispectral cameras have light sensors to calibrate light conditions, saturation issues can still be found in UAV images. For example, with a downwelling light sensor (DLS), which is a 5-band sensor that connects to the multispectral camera, the Rededge M can measure the ambient light during a flight and record the light information in the images. After the camera calibration, the information detected by the DLS can be used to correct lighting changes during a flight, which usually happens because the clouds cover the sun during a UAV flight. The clouds are believed to affect the saturation issues, even though sunshine is supposed to correct reflectance for real-time conditions. Saturated values decrease the quality of the data. The retrieval of required indexes, such as NDVI and LAI, are important for the estimation of soil heat flux *G* and sensible heat flux *H*.

Another issue for ET estimation with UAVs is the bidirectional reflectance distribution function (BRDF) effects. For many UAV applications, the reflectance model for canopy measurements is simplified to assume a strict nadir (or straight down) viewing angle and a static illumination source [125,129,130]. However, this assumption does not consider the bidirectional reflectance distribution function (BRDF). The BRDF is a function of wavelength, observer azimuth, observer zenith, illumination azimuth, and illumination zenith [129]. In satellite images, the effect of BRDF is relatively uniform because the satellite covers a wide region in a single frame. However, this simplification is not valid for UAV platforms equipped with an imaging system with a wide field-of-view (FOV).

Further experiments should be based on multispectral measurements, and UAV image acquisition should be conducted to select those spectral bands which are most useful and sensitive for ET estimations. Cameras should be designed only for those needed bands, which will lower the cost of the sensors. The availability of low-cost UAV platforms and specialized cameras will also make the UAVs’ application to ET estimation more competitive.

#### 4.2.3. Thermal Camera Calibration and Image Processing

The thermal images from UAVs are becoming a useful source for ET estimations because of their higher temporal and spatial resolution compared with those obtained from the satellites. The thermal camera has a spectral response from 7 μm to 14 μm. The accuracy can be as high as ±1 ${}^{\circ }$C. Thermal remote sensing images were first used in 1973 to estimate ET [131]. Temperature information is usually converted into land surface characteristics such as albedo, LAI, and surface emissivity. The TIR band is considered as the most critical variable for estimating the sensible heat flux and ground heat flux [33]. The cooled thermal cameras are usually more sensitive and accurate than uncooled thermal cameras [132]. However, cooled thermal cameras are very big, expensive, and energy-consuming [47]. Therefore, cooled thermal cameras can hardly be used on UAV platforms. In contrast, the uncooled thermal cameras are usually lighter [130], which are usually less than 200 g, low power consumption [133], and less expensive than cooled thermal cameras.

The thermal camera has many advantages, though its microbolometer is not always sensitive and accurate [47]. Most thermal cameras are not always calibrated, so can only measure the relative temperature instead of the absolute values. In precision agriculture, it is necessary to measure the absolute temperature in many applications [130], such as crop monitoring [134], pest detection [135], and disease detection [136]. Unstable outdoor environmental factors can also cause serious measurement drift during flight missions. Post-processing like mosaicking might further lead to measurement errors. To solve these two fundamental problems, in [46], the authors conducted three experiments to research the best practices of thermal image collection using UAVs. To calibrate TIR images, in [16], Park et al. used the water body and rubber plates as cold and hot features. IR Flash Version 2 is usually used to process thermal UAV images for image format transformation.

The correlation between the measured IR temperature from calibration boards and the estimates by thermal cameras were found to be unacceptable sometimes. Without warming up the uncooled thermal camera, the temperature difference between the thermal camera and calibration board can be as high as ±10 ${}^{\circ }$C. The land surface temperature is the most important data for SEBAL and the estimation of surface energy fluxes; thus, its accuracy is the key for a reliable ET estimation.

Many researchers also focus on thermal camera calibration issues. For example, [47] proposed a new calibration algorithm based on neural networks. The calibration algorithms considered the thermal camera temperature and the digital response of the microbolometer as input data. Based on the calibration result, the accuracy increased from 3.55 to 1.37 ${}^{\circ }$C. In [45], Torres-Rua et al. presented a vicarious calibration methodology (UAV-specific, time-specific, flight-specific, and sensor-specific) for thermal camera images traceable back to NIST-standards and current atmospheric correction methods.

For future research, uncooled thermal cameras can be used to evaluate with other temperatures sensor information to acquire reliable thermal information, such as atmospherically corrected satellite images and temperature canopy sensors.

#### 4.2.4. Images Stitching and Orthomosaic Image Generation

After UAV images are collected, all of the aerial images need to be stitched together to generate the orthomosaic images (Appendix A.1 and Appendix A.2). Some problems are identified when creating mosaics, such as fault lines, blurriness, and replicated features, especially with the thermal data. To overcome the thermal camera’s effect, a higher overlap for the thermal camera can be a good choice. With an increase in the image overlap by 5%, most of the fuzziness and replicated problems were eliminated [37].

There are many types of software that can be used for image stitching, such as Pix4D (Pix4D, Prilly, Switzerland), Agisoft Metashape, RealityCapture, and DroneDeploy (DroneDeploy, San Francisco, CA, USA). For example, during the image stitching workflow using the Agisoft Metashape, there are several steps for image processing, which include aligning photos, optimize cameras, build mesh, build dense cloud, build digital elevation model (DEM), and generating orthomosaic.

## 5. Conclusions

As a new remote sensing platform, researchers are more and more interested in the potential of UAVs in precision agriculture. Compared with traditional remote sensing platforms, the UAVs can be more flexible in the field. For example, UAVs can be operated at any time if the weather is within the operating limitations. The satellite has a fixed flight path, UAVs are mobile and flexible for site selection. Mounted on the UAVs, lightweight sensors, such as RGB cameras, multispectral cameras, and thermal infrared cameras, can be used to collect high-resolution images. While there are many advantages with using UAVs, there are still challenges for UAVs when used for estimating ET. Many researchers fly the UAVs at different height, using specialized equipment and relying on data analysis expertise. As researchers try to understand and realize the potential of the UAVs for ET estimation, efficient workflow, image processing, and better software are still under developing.

ET estimation methods and related agricultural applications have been well-developed over the past decades. Although remote sensing ET models can help obtain relatively accurate spatially distributed ET data, many questions still remain. As discussed in this article, each ET estimation model has its advantages and disadvantages. For example, METRIC/SEBAL methods are more recognized by the remote sensing researchers, but they are based on satellite (Landsat) platforms. Significant modifications would be required to make them work with UAV images. The TSEB model is less widely known, but it offers more potential for UAV applications in many crop conditions, especially tree crops such as almonds, pomegranates, or peaches. When flying a UAV, weather conditions, field-size, flight time, and many other factors should also be considered when choosing the appropriate algorithms for ET estimation.

No existing methods can fully satisfy the spatial, temporal, spectral, and accuracy requirements for ET-based science and applications. Therefore, innovative methods or models for ET estimation are required by using UAVs. There are five requirements to map ET with high fidelity in the future [137], which are high frequency, high spatial resolution, high temporal resolution, large spatial coverage, and long-term monitoring. High frequency will improve the differentiation of water stress between crops, which enables more efficient water management. High spatial resolution can help detect spatially heterogeneous responses to water stress. As ET is highly variable within and among days, high temporal resolution can help detect crops ET in real-time. Large spatial coverage can help detect large scale drought. Long term monitoring will be important to record ET variability overtime.

Compared with other satellite-based remote sensing methods, the UAV platform and lightweight sensors can provide better quality, higher spatial, and temporal resolution images. The UAVs can be used to estimate ET on an excellent spatial scale and with flexible flight schedules. In the future, (1) the TSEB and DTD models have great potential for ET estimation, since they can separate the soil and canopy with high-resolution UAV imagery; (2) taking advantage of the UAV high-resolution imagery, research related to individual tree-level ET estimation will be possible and useful for analyzing the temporal and spatial variability of the crops in the field; (3) deep learning algorithms can be used for processing high-resolution UAV imagery, such as individual tree-level canopy or soil segmentation; (4) our research results [138,139] show that there is strong correlation between the NDVI and crop coefficient at individual tree-level ET estimation. Further study can be conducted to create new vegetation indexes using machine learning and deep learning algorithms.

## Figures and Tables

**Table 1:** Evapotranspiration (ET) estimation using UAV platforms.

Study Sites	UAV Platforms	Sensors	Method	Crops	References
Ames, Iowa, USA	eBee Ag	Sequoia, Cannon S110 thermoMAP camera	SEBAL	Corn and soybean	[32]
Scipio, UT Lodi, CA, USA	AggieAir	Canno S-95 ICI thermal camera	METRIC	Vineyard	[33]
Pinto Bandeira city Rio Grande do Sul State, Brazil	AIBOTIX	Nikon CoolpixA Camera	METRIC	Vineyard	[34]
HOBE agricultural site, Denmark	Q300, QuestUAV	Optris PI 450	TSEB	Barley	[21]
Lodi, CA, USA	Cessna TU206	ImperX Bobcat B8430 ThermaCAM SC640	TSEB	Vineyard	[35]
Lodi, CA, USA	AggieAir	NA	TSEB	Vineyard	[36]
Pinto Bandeira Serra Gaucha, Brazil	AIBOTIX Hexacoptero	Nikon CoolpixA camera	TSEB	Vineyard	[37]
Lodi, CA, USA	NA	NA	TSEB	Vineyard	[38]
Pencahue Valley Región del Maule, Chile	NA	Mini MCA-6 EasIR-9	TSEB	Olive	[31]
Bushland, Texas, USA	AggieAir	Kodak1 thermal infrared model 760	TSEB	Sorghum and corn	[39]
Petit-Nobressart, Luxembourg	MikroKopter OktoXL	Samsung ES80 Optris Pi 400	TSEB	Grassland	[40]
Lodi, CA, USA	Cessna TU206	ImperX Bobcat B8430 ThermaCAM SC640	OSEB	Vineyard	[35]
Petit-Nobressart, Luxembourg	MikroKopter OktoXL	Samsung ES80 Optris Pi 400	OSEB	Grassland	[40]
Tatura, Victoria, Australia	DJI S1000	A65 and RedEdge M	HRMET	Peach, nectarine, and corn	[16]

NA indicates not available.

**Table 2:** Multispectral and thermal infrared sensors on UAV platforms.

Sensor	Function	Resolution	Weights	Dimensions	Spectral Bands	Accuracy
Rededge M	Multispectral	1280 × 960 pixels	231.9 g	8.7 × 5.9 × 4.54 cm	Blue, green, red, red edge, near infrared (NIR)	8.2 cm/pixel, per band at 120 m
MAPIR Survey 3	Multispectral	4608 × 3456 pixels	76 g	5.9 × 4.15 × 3.6 cm	375–650 nm	4.05 cm/pixel at 120 m
Mini MCA-6	Multispectral	1280 × 1024 pixels	700 g	13.14 × 7.83 × 8.76 cm	450–1000 nm	3.3 cm/pixel at 60 m
Tetracam ADC Lite	Multispectral	2048 × 1536 pixels	200 g	11.4 × 7.7 × 6.05 cm	Red, green, NIR	5 cm/pixel at 150 m
Sequoia	Multispectral	4608 × 3456 pixels	72 g	5.9 × 4.1 × 2.8 cm	Green, red, red edge and near infrared	17 cm/pixel at 100 m
Cannon S 110	Near infrared	4000 × 3000 pixels	198 g	9.9 × 5.9 × 2.7 cm	NIR	3.5 cm/pixel at 100 m
ICI SWIR 640 P	Short-wave infrared	640 × 512 pixel	130 g	4.6 × 4.6 × 2.95 cm	0.9–1.7 μm	±1 ${}^{\circ }$C
ICI 9640 P	Thermal infrared	640 × 480 pixel	37 g	3.4 × 3.0 × 3.4 cm	7–14 μm	±1 ${}^{\circ }$C
ICI 8640 P	Thermal infrared	640 × 480 pixel	74.5 g	4.5 × 4.5 × 3.9 cm	7–14 μm	±1 ${}^{\circ }$C
FLIR Vue Pro R 640	Thermal infrared	640 × 512 pixel	72 g	5.74 × 4.44 cm	7.5–13.5 μm	±5 ${}^{\circ }$C
Optris PI 450	Thermal infrared	382 × 288 pixels	240 g	4.6 × 5.6 × 6.8 cm	8–14 μm	±2 ${}^{\circ }$C or ±2%
ThermalCAM SC640	Thermal infrared	640 × 480 pixel	1.7 kg	28.2 × 14.4 × 14.7 cm	7.5–13 μm	±2 ${}^{\circ }$C or ±2%
EasIR-9	Thermal infrared	288 × 384 pixel	1 kg	11.2 × 18.2 × 25.2 cm	8–14 μm	±2 ${}^{\circ }$C or ±2%
thermoMAP	Thermal infrared	640 × 512 pixel	134 g	56 × 46 × 26 cm	7–15 μm	±5 ${}^{\circ }$C
A65	Thermal infrared	640 × 512 pixel	200 g	29.5 × 20.0 × 10.5 cm	7.5–13 μm	±5 ${}^{\circ }$C
Optris PI 400	Thermal infrared	382 × 288 pixel	240 g	4.6 × 5.6 × 6.8 cm	8–14 μm	±2 ${}^{\circ }$C

**Table 3:** Comparisons of the different ET estimation methods.

Methods	Applications with UAVs	Advantages	Disadvantages
OSEB	Vineyard [35], grassland [40]	(1) Treat the surface as big leaf and therefore as a simple uniform layer.	(1) Uses empirical parameters to explain differences in the aerodynamic and radiometric components; (2) Assumes the whole surface as a uniform layer, which does not take advantage of UAV high-resolution imagery; (3) Less sensitive to land surface temperature variations than the TSEB model.
HRMET	Peach, nectarine [16], and corn	(1) Only requires basic meteorological data, spatial surface temperature, and canopy structure data; (2) Does not depend on wet and dry reference features to calculate turbulent fluxes.	(1) Needs more validation for clumped canopy structure, such as trees and vines.
ML/ANN	Vineyard [56]	(1) Capture non-linear crop characteristics	(1) Requires large amount of data for training models and validation
TSEB	Barley [21], vineyard [35,36,37,38], olive [31], sorghum and corn [39], grassland [40]	(1) The calculation of sensible heat flux and latent heat flux for canopy and soil are separate; (2) Parameterization of resistances is easier compared with a single layer model	(1) Sensitive to the temperature difference between the land surface and air; (2) The measurement of the absolute land surface temperature is inaccurate
DTD	Barley [21], corn and soybean	(1) One more input dataset, the land surface temperature retrieved one hour after sunrise; (2) Minimizes the bias in the temperature estimation; (3) Separates the land surface temperature into vegetation and soil temperatures	(1) Requires flights at two times during the morning hours, thus complicating flight missions
SEBAL	Corn and soybean [32]	(1) Requires minimum ground-based data; (2) Automatic internal correction	(1) Selecting hot or cold pixels is subjective, which can cause variations in ET estimation
METRIC	Vineyard [33,34]	(1) Eliminates the need for absolute surface temperature calibration; (2) Requires minimum ground-based data; (3) Automatic internal correction	(1) Selecting hot or cold pixels is subjective, which can cause variations in ET estimation

**Table 4:** Comparisons of the different ET estimation methods with UAVs.

Methods	Applications with UAVs	Accuracy of $R_{n}$	Accuracy of *G*	Accuracy of LE	Accuracy of *H*
OSEB TSEB	Grassland [40]	$R^{2}$ of 0.98 $R^{2}$ of 0.99	$R^{2}$ of 0.73 $R^{2}$ of 0.83	$R^{2}$ of 0.92 $R^{2}$ of 0.93	$R^{2}$ of 0.79 $R^{2}$ of 0.84
TSEB DTD	Barley [21]	RMSE of 44 W m${}^{-2}$ RMSE of 44 W m${}^{-2}$	RSME of 38 W m${}^{-2}$ RSME of 48 W m${}^{-2}$	RMSE of 94 W m${}^{-2}$ RSME of 67 W m${}^{-2}$	RMSE of 85 W m${}^{-2}$ RSME of 59 W m${}^{-2}$
TSEB DATTUTDUT	Vineyard [35]	RMSE of 33 W m${}^{-2}$ RMSE of 66 W m${}^{-2}$	RSME of 33 W m${}^{-2}$ RSME of 40 W m${}^{-2}$	RMSE of 87 W m${}^{-2}$ RSME of 150 W m${}^{-2}$	RMSE of 42 W m${}^{-2}$ RSME of 68 W m${}^{-2}$
TSEB	Olive [31]	RMSE of 38 W m${}^{-2}$	RMSE of 19 W m${}^{-2}$	RMSE of 50 W m${}^{-2}$	RMSE of 56 W m${}^{-2}$
SEBAL	Corn, soybean [32]	$R^{2}$ of 0.71	$R^{2}$ of 0.17 and 0.22	$R^{2}$ of 0.82	$R^{2}$ of 0.5

## Abbreviations

The following abbreviations are used in this manuscript:

MDPI	Multidisciplinary Digital Publishing Institute
ET	Evapotranspiration
EC	Eddy covariance
UAVs	Unmanned Aerial Vehicles
METRIC	Mapping Evapotranspiration at high Resolution with Internalized Calibration
NDVI	Normalized Difference Vegetation Index
DTD	Dual Temperature Difference
SEBAL	Surface Energy Balance Algorithm for Land
RGB	Red Green and Blue
RSEB	Remote Sensing Energy Balance
OSEB	One Source Energy Balance
DATTUTDUT	Deriving atmosphere turbulent transport useful to dummies using temperature
HRMET	High Resolution Mapping of Evapotranspiration
ML	Machine Learning
ANN	Artificial Neural Networks
ELM	Extreme Learning Machine
SVM	Support Vector Machine
ANFIS	Adaptive Neuro-fuzzy Inference System
TSEB	Two source energy balance
TSEB-PT	Priestley–Taylor Two Source Energy Balance
LAI	Leaf Area Index
CWSI	Crop Water Stress Index
S-SEBI	Simplified Surface Energy Balance Index
SEBI	Surface Energy Balance Index
SEBS	Surface Energy Balance System
DLS	Downwelling Light Sensor
TIR	Thermal infrared
LST	Land Surface Temperature
FOV	Field of View
NIR	Near Infrared
JPEG	Joint Photographic Experts Group
SWIR	Short-wave infrared

## Appendix A. MATLAB Code and Photoscan Settings

### Appendix A.1. Matlab Code for Calculating Drone Image NDVI

The Matlab code is attached here for calculating NDVI of drone images, which can be found at Github: https://github.com/niuhaoyu16/NDVI-for-drone-images.

### Appendix A.2. Agisoft Photoscan Image Processing Settings

Agisoft Photoscan image processing settings is available at Github: https://github.com/niuhaoyu16/AgisoftPhotoscan-Images-processing-settings.

## References

[1] Verstraeten W., Veroustraete F., Feyen J. (2008). Assessment of Evapotranspiration and Soil Moisture Content across Different Scales of Observation. Sensors.

[2] Wu J., Wang D. (2005). Estimating Evaporation Coefficient during Two-stage Evaporation from Soil Surfaces. Soil Sci..

[3] Kaplan S., Myint S.W., Fan C., Brazel A.J. (2014). Quantifying Outdoor Water Consumption of Urban Land Use/Land Cover: Sensitivity to Drought. Environ. Manag..

[4] Wetzel P.J., Chang J.T. (1988). Evapotranspiration from Nonuniform Surfaces: A First Approach for Short-Term Numerical Weather Prediction. Mon. Weather Rev..

[5] Allen R.G., Pereira L.S., Raes D., Smith M. (1998). FAO Irrigation and Drainage Paper No. 56. Rome Food Agric. Organ. U. N..

[6] Xu C.Y., Singh V. (2005). Evaluation of Three Complementary Relationship Evapotranspiration Models by Water Balance Approach to Estimate Actual Regional Evapotranspiration in Different Climatic Regions. J. Hydrol..

[7] Liou Y.A., Kar S. (2014). Evapotranspiration Estimation with Remote Sensing and Various Surface Energy Balance Algorithms—A Review. Energies.

[8] Allen R., Irmak A., Trezza R., Hendrickx J.M., Bastiaanssen W., Kjaersgaard J. (2011). Satellite-based ET Estimation in Agriculture Using SEBAL and METRIC. Hydrol. Process..

[9] Angus D., Watts P. (1984). Evapotranspiration-How Good is the Bowen Ratio Method?. Developments in Agricultural and Managed Forest Ecology.

[10] Fritschen L.J. (1965). Accuracy of Evapotranspiration Determinations by the Bowen Ratio Method. Hydrol. Sci. J..

[11] Nagler P.L., Scott R.L., Westenburg C., Cleverly J.R., Glenn E.P., Huete A.R. (2005). Evapotranspiration on Western US Rivers Estimated Using the Enhanced Vegetation Index from MODIS and Data from Eddy Covariance and Bowen Ratio Flux Towers. Remote Sens. Environ..

[12] Su Z. (2002). The Surface Energy Balance System (SEBS) for Estimation of Turbulent Heat Fluxes. Hydrol. Earth Syst. Sci..

[13] Kustas W., Anderson M. (2009). Advances in Thermal Infrared Remote Sensing for Land Surface Modeling. Agric. For. Meteorol..

[14] Allen R.G., Tasumi M., Morse A., Trezza R. (2005). A Landsat-based Energy Balance and Evapotranspiration Model in Western US Water Rights Regulation and Planning. Irrig. Drain. Syst..

[15] Bastiaanssen W.G., Menenti M., Feddes R., Holtslag A. (1998). A Remote Sensing Surface Energy Balance Algorithm for Land (SEBAL). 1. Formulation. J. Hydrol..

[16] Park S. (2018). Estimating Plant Water Stress and Evapotranspiration Using Very-high-resolution (VHR) UAV Imagery. Ph.D. Thesis.

[17] Kustas W.P., Norman J.M. (1997). A Two-source Approach for Estimating Turbulent Fluxes Using Multiple Angle Thermal Infrared Observations. Water Resour. Res..

[18] McShane R.R., Driscoll K.P., Sando R. (2017). A Review of Surface Energy Balance Models for Estimating Actual Evapotranspiration with Remote Sensing at High Spatio Temporal Resolution over Large Extents. Scientific Investigations Report 2017–5087.

[19] Allen R.G., Tasumi M., Trezza R. (2007). Satellite-based Energy Balance for Mapping Evapotranspiration with Internalized Calibration (METRIC)—Model. J. Irrig. Drain. Eng..

[20] Norman J.M., Kustas W.P., Humes K.S. (1995). Source Approach for Estimating Soil and Vegetation Energy Fluxes in Observations of Directional Radiometric Surface Temperature. Agric. For. Meteorol..

[21] Hoffmann H., Nieto H., Jensen R., Guzinski R., Zarco-Tejada P., Friborg T. (2016). Estimating Evaporation with Thermal UAV Data and Two-source Energy Balance Models. Hydrol. Earth Syst. Sci..

[22] Quattrochi D.A., Luvall J.C. (1999). Thermal Infrared Remote Sensing for Analysis of Landscape Ecological Processes: Methods and Applications. Landsc. Ecol..

[23] Moran M.S., Jackson R.D. (1991). Assessing the Spatial Distribution of Evapotranspiration Using Remotely Sensed Inputs. J. Environ. Qual..

[24] Kustas W., Norman J. (1996). Use of Remote Sensing for Evapotranspiration Monitoring over Land Surfaces. Hydrol. Sci. J..

[25] Díaz-Varela R., de la Rosa R., León L., Zarco-Tejada P. (2015). High-resolution Airborne UAV Imagery to Assess Olive Tree Crown Parameters Using 3D Photo Reconstruction: Application in Breeding Trials. Remote Sens..

[26] Gonzalez-Dugo V., Goldhamer D., Zarco-Tejada P.J., Fereres E. (2015). Improving the Precision of Irrigation in a Pistachio Farm Using an Unmanned Airborne Thermal System. Irrig. Sci..

[27] Swain K.C., Thomson S.J., Jayasuriya H.P. (2010). Adoption of an Unmanned Helicopter for Low-altitude Remote Sensing to Estimate Yield and Total Biomass of a Rice Crop. Trans. ASABE.

[28] Zarco-Tejada P.J., González-Dugo V., Williams L., Suárez L., Berni J.A., Goldhamer D., Fereres E. (2013). A PRI-based Water Stress Index Combining Structural and Chlorophyll Effects: Assessment Using Diurnal Narrow-band Airborne Imagery and the CWSI Thermal Index. Remote Sens. Environ..

[29] Zhao T., Chen Y., Ray A., Doll D. Quantifying almond water stress using unmanned aerial vehicles (UAVs): Correlation of stem water potential and higher order moments of non-normalized canopy distribution. Proceedings of the ASME 2017 International Design Engineering Technical Conferences and Computers and Information in Engineering Conference.

[30] Zhao T., Niu H., de la Rosa E., Doll D., Wang D., Chen Y. (2018). Tree canopy differentiation using instance-aware semantic segmentation. Proceedings of the 2018 ASABE Annual International Meeting.

[31] Ortega-Farías S., Ortega-Salazar S., Poblete T., Kilic A., Allen R., Poblete-Echeverría C., Ahumada-Orellana L., Zuñiga M., Sepúlveda D. (2016). Estimation of Energy Balance Components over a Drip-irrigated Olive Orchard Using Thermal and Multispectral Cameras Placed on a Helicopter-based Unmanned Aerial Vehicle (UAV). Remote Sens..

[32] Montibeller Á.G. (2017). Estimating Energy Fluxes and Evapotranspiration of Corn and Soybean with an Unmanned Aircraft System in Ames, Iowa. Master’s Thesis.

[33] Elarab M. (2016). The Application of Unmanned Aerial Vehicle to Precision Agriculture: Chlorophyll, Nitrogen, and Evapotranspiration Estimation. Ph.D. Thesis.

[34] dos Reis T., Monteiro R., Garcia E., Albuquerque M., Espinoza J., Ferreira J. Actual Evapotranspiration Estimated by Orbital Sensors, UAV and Meteorological Station for Vineyards in the Southern Brazil. Proceedings of the IV Inovagri International Meeting.

[35] Xia T., Kustas W.P., Anderson M.C., Alfieri J.G., Gao F., McKee L., Prueger J.H., Geli H.M., Neale C.M., Sanchez L. (2016). Mapping Evapotranspiration with High-resolution Aircraft Imagery over Vineyards Using One-and Two-source Modeling Schemes. Hydrol. Earth Syst. Sci..

[36] Nieto H., Bellvert J., Kustas W.P., Alfieri J.G., Gao F., Prueger J., Torres-Rua A., Hipps L.E., Elarab M., Song L. Unmanned airborne thermal and mutilspectral imagery for estimating evapotranspiration in irrigated vineyards. Proceedings of the 2017 IEEE International Geoscience and Remote Sensing Symposium (IGARSS).

[37] Monteiro C. (2019). Evapotranspiration Estimate Using Energy Balance Two Source Model With UAV Images: A Study in Vineyard. Am. J. Eng. Res..

[38] Nieto H., Kustas W.P., Torres-Rúa A., Alfieri J.G., Gao F., Anderson M.C., White W.A., Song L., del Mar Alsina M., Prueger J.H. (2019). Evaluation of TSEB Turbulent Fluxes Using Different Methods for the Retrieval of Soil and Canopy Component Temperatures from UAV Thermal and Multispectral Imagery. Irrig. Sci..

[39] Chávez J.L., Gowda P., Howell T., Neale C., Copeland K. (2009). Estimating Hourly Crop ET Using a Two-source Energy Balance Model and Multispectral Airborne Imagery. Irrig. Sci..

[40] Brenner C., Thiem C.E., Wizemann H.D., Bernhardt M., Schulz K. (2017). Estimating Spatially Distributed Turbulent Heat Fluxes from High-resolution Thermal Imagery Acquired with a UAV System. Int. J. Remote Sens..

[41] Menenti M., Bolle H.J., Feddes R.A., Kalma J.D. (1993). Parameteraization of Land Surface Evaporation by Means of Location Dependent Potential Evaporation and Surface Temperature Range. Exchange Processes at the Land Surface for a Range of Space and Time Scales.

[42] Jackson R.D., Idso S., Reginato R., Pinter P. (1981). Canopy Temperature as a Crop Water Stress Indicator. Water Resour. Res..

[43] Roerink G., Su Z., Menenti M. (2000). S-SEBI: A Simple Remote Sensing Algorithm to Estimate the Surface Energy Balance. Phys. Chem. Earth Part B Hydrol. Ocean. Atmos..

[44] Zhao T., Yang Y., Niu H., Wang D., Chen Y. Comparing U-Net convolutional network with mask R-CNN in the performances of pomegranate tree canopy segmentation. Proceedings of the Multispectral, Hyperspectral, and Ultraspectral Remote Sensing Technology, Techniques and Applications VII.

[45] Torres-Rua A. (2017). Vicarious Calibration of sUAS Microbolometer Temperature Imagery for Estimation of Radiometric Land Surface Temperature. Sensors.

[46] Zhao T., Niu H., Anderson A., Chen Y., Viers J. A detailed study on accuracy of uncooled thermal cameras by exploring the data collection workflow. Proceedings of the Autonomous Air and Ground Sensing Systems for Agricultural Optimization and Phenotyping III.

[47] Ribeiro-Gomes K., Hernández-López D., Ortega J.F., Ballesteros R., Poblete T., Moreno M.A. (2017). Uncooled Thermal Camera Calibration and Optimization of the Photogrammetry Process for UAV Applications in Agriculture. Sensors.

[48] Niu H., Zhao T., Wang D., Chen Y. A UAV resolution and waveband aware path planning for onion irrigation treatments inference. Proceedings of the 2019 International Conference on Unmanned Aircraft Systems (ICUAS).

[49] Zipper S.C., Loheide S.P. (2014). Using Evapotranspiration to Assess Drought Sensitivity on a Subfield Scale with HRMET, a High Resolution Surface Energy Balance Model. Agric. For. Meteorol..

[50] Hsu K.l., Gupta H.V., Sorooshian S. (1995). Artificial Neural Network Modeling of the Rainfall-runoff Process. Water Resour. Res..

[51] Abrahart R.J., Anctil F., Coulibaly P., Dawson C.W., Mount N.J., See L.M., Shamseldin A.Y., Solomatine D.P., Toth E., Wilby R.L. (2012). Two Decades of Anarchy? Emerging Themes and Outstanding Challenges for Neural Network River Forecasting. Prog. Phys. Geogr..

[52] Keshtegar B., Piri J., Kisi O. (2016). A Nonlinear Mathematical Modeling of Daily Pan Evaporation Based on Conjugate Gradient Method. Comput. Electron. Agric..

[53] Kousari M.R., Hosseini M.E., Ahani H., Hakimelahi H. (2017). Introducing an Operational Method to Forecast Long-term Regional Drought Based on the Application of Artificial Intelligence Capabilities. Theor. Appl. Climatol..

[54] Moghaddamnia A., Gousheh M.G., Piri J., Amin S., Han D. (2009). Evaporation Estimation Using Artificial Neural Networks and Adaptive Neuro-fuzzy Inference System Techniques. Adv. Water Resour..

[55] Park S., Im J., Jang E., Rhee J. (2016). Drought Assessment and Monitoring through Blending of Multi-sensor Indices Using Machine Learning Approaches for Different Climate Regions. Agric. For. Meteorol..

[56] Poblete T., Ortega-Farías S., Moreno M., Bardeen M. (2017). Artificial Neural Network to Predict Vine Water Status Spatial Variability Using Multispectral Information Obtained from an Unmanned Aerial Vehicle (UAV). Sensors.

[57] Norman J., Kustas W., Prueger J., Diak G. (2000). Surface Flux Estimation Using Radiometric Temperature: A Dual-temperature-difference Method to Minimize Measurement Errors. Water Resour. Res..

[58] Boulet G., Olioso A., Ceschia E., Marloie O., Coudert B., Rivalland V., Chirouze J., Chehbouni G. (2012). An Empirical Expression to Relate Aerodynamic and Surface Temperatures for Use within Single-source Energy Balance Models. Agric. For. Meteorol..

[59] Kalma J., Jupp D. (1990). Estimating Evaporation from Pasture Using Infrared Thermometry: Evaluation of a One-layer Resistance Model. Agric. For. Meteorol..

[60] Massman W. (1999). A Model Study of kBH-1 for Vegetated Surfaces Using Localized Near-field Lagrangian Theory. J. Hydrol..

[61] Verhoef A., De Bruin H., Van Den Hurk B. (1997). Some Practical Notes on the Parameter kB-1 for Sparse Vegetation. J. Appl. Meteorol..

[62] Colaizzi P.D., Evett S.R., Howell T.A., Tolk J.A. Comparison of aerodynamic and radiometric surface temperature using precision weighing lysimeters. Proceedings of the Remote Sensing and Modeling of Ecosystems for Sustainability.

[63] Norman J.M., Becker F. (1995). Terminology in Thermal Infrared Remote Sensing of Natural Surfaces. Agric. For. Meteorol..

[64] Kustas W.P., Norman J.M. (1999). Evaluation of Soil and Vegetation Heat Flux Predictions Using a Simple Two-source Model with Radiometric Temperatures for Partial Canopy Cover. Agric. For. Meteorol..

[65] Colaizzi P.D., Kustas W.P., Anderson M.C., Agam N., Tolk J.A., Evett S.R., Howell T.A., Gowda P.H., O’Shaughnessy S.A. (2012). Two-source Energy Balance Model Estimates of Evapotranspiration Using Component and Composite Surface Temperatures. Adv. Water Resour..

[66] Timmermans W.J., Kustas W.P., Anderson M.C., French A.N. (2007). An Intercomparison of the Surface Energy Balance Algorithm for Land (SEBAL) and the Two-source Energy Balance (TSEB) Modeling Schemes. Remote Sens. Environ..

[67] Troufleau D., Lhomme J.P., Monteny B., Vidal A. (1997). Sensible Heat Flux and Radiometric Surface Temperature over Sparse Sahelian Vegetation. I. An Experimental Analysis of the kB-1 Parameter. J. Hydrol..

[68] Matsushima D. (2005). Relations between Aerodynamic Parameters of Heat Transfer and Thermal-infrared Thermometry in the Bulk Surface Formulation. J. Meteorol. Soc. Jpn. Ser. II.

[69] Brutsaert W. (2005). Hydrology: An Introduction.

[70] Timmermans W.J., Kustas W.P., Andreu A. (2015). Utility of an Automated Thermal-based Approach for Monitoring Evapotranspiration. Acta Geophys..

[71] Crisci C., Ghattas B., Perera G. (2012). A Review of Supervised Machine Learning Algorithms and Their Applications to Ecological Data. Ecol. Model..

[72] Antonopoulos V.Z., Gianniou S.K., Antonopoulos A.V. (2016). Artificial Neural Networks and Empirical Equations to Estimate Daily Evaporation: Application to Lake Vegoritis, Greece. Hydrol. Sci. J..

[73] Gocić M., Motamedi S., Shamshirband S., Petković D., Ch S., Hashim R., Arif M. (2015). Soft Computing Approaches for Forecasting Reference Evapotranspiration. Comput. Electron. Agric..

[74] Kisi O., Sanikhani H., Zounemat-Kermani M., Niazi F. (2015). Long-term Monthly Evapotranspiration Modeling by Several Data-driven Methods without Climatic Data. Comput. Electron. Agric..

[75] Mehdizadeh S., Behmanesh J., Khalili K. (2017). Using MARS, SVM, GEP and Empirical Equations for Estimation of Monthly Mean Reference Evapotranspiration. Comput. Electron. Agric..

[76] Misaghian N., Shamshirband S., Petković D., Gocic M., Mohammadi K. (2017). Predicting the Reference Evapotranspiration Based on Tensor Decomposition. Theor. Appl. Climatol..

[77] Petković D., Gocic M., Shamshirband S., Qasem S.N., Trajkovic S. (2016). Particle Swarm Optimization-based Radial Basis Function Network for Estimation of Reference Evapotranspiration. Theor. Appl. Climatol..

[78] Tabari H., Martinez C., Ezani A., Talaee P.H. (2013). Applicability of Support Vector Machines and Adaptive Neurofuzzy Inference System for Modeling Potato Crop Evapotranspiration. Irrig. Sci..

[79] Yassin M.A., Alazba A.A., Mattar M.A. (2016). Comparison between Gene Expression Programming and Traditional Models for Estimating Evapotranspiration under Hyper Arid Conditions. Water Resour..

[80] Dou X., Yang Y. (2018). Evapotranspiration Estimation Using Four Different Machine Learning Approaches in Different Terrestrial Ecosystems. Comput. Electron. Agric..

[81] Gocic M., Petković D., Shamshirband S., Kamsin A. (2016). Comparative Analysis of Reference Evapotranspiration Equations Modelling by Extreme Learning Machine. Comput. Electron. Agric..

[82] Hashim R., Roy C., Motamedi S., Shamshirband S., Petković D., Gocic M., Lee S.C. (2016). Selection of Meteorological Parameters Affecting Rainfall Estimation Using Neuro-fuzzy Computing Methodology. Atmos. Res..

[83] Petković D., Gocic M., Trajkovic S., Shamshirband S., Motamedi S., Hashim R., Bonakdari H. (2015). Determination of the Most Influential Weather Parameters on Reference Evapotranspiration by Adaptive Neuro-fuzzy Methodology. Comput. Electron. Agric..

[84] Alizadeh M.J., Kavianpour M.R., Kisi O., Nourani V. (2017). A New Approach for Simulating and Forecasting the Rainfall-runoff Process within the Next Two Months. J. Hydrol..

[85] Shamshirband S., Amirmojahedi M., Gocić M., Akib S., Petković D., Piri J., Trajkovic S. (2015). Estimation of Reference Evapotranspiration Using Neural Networks and Cuckoo Search Algorithm. J. Irrig. Drain. Eng..

[86] Pour-Ali Baba A., Shiri J., Kisi O., Fard A.F., Kim S., Amini R. (2012). Estimating Daily Reference Evapotranspiration Using Available and Estimated Climatic Data by Adaptive Neuro-fuzzy Inference System (ANFIS) and Artificial Neural Network (ANN). Hydrol. Res..

[87] Torres-Rua A.F., Ticlavilca A.M., Aboutalebi M., Nieto H., Alsina M.M., White A., Prueger J.H., Alfieri J.G., Hipps L.E., McKee L.G. Estimation of evapotranspiration and energy fluxes using a deep learning-based high-resolution emissivity model and the two-source energy balance model with sUAS information. Proceedings of the Autonomous Air and Ground Sensing Systems for Agricultural Optimization and Phenotyping V.

[88] Kustas W.P., Norman J.M. (2000). A Two-source Energy Balance Approach Using Directional Radiometric Temperature Observations for Sparse Canopy Covered Surfaces. Agron. J..

[89] Priestley C.H.B., Taylor R. (1972). On the Assessment of Surface Heat Flux and Evaporation Using Large-scale Parameters. Mon. Weather Rev..

[90] French A.N., Hunsaker D.J., Thorp K.R. (2015). Remote Sensing of Evapotranspiration over Cotton Using the TSEB and METRIC Energy Balance Models. Remote Sens. Environ..

[91] Choi M., Kustas W.P., Anderson M.C., Allen R.G., Li F., Kjaersgaard J.H. (2009). An Intercomparison of Three Remote Sensing-based Surface Energy Balance Algorithms over a Corn and Soybean Production Region (Iowa, US) during SMACEX. Agric. For. Meteorol..

[92] Colaizzi P., Evett S., Howell T., Li F., Kustas W., Anderson M. (2012). Radiation Model for Row Crops: I. Geometric View Factors and Parameter Optimization. Agron. J..

[93] Song L., Liu S., Kustas W.P., Zhou J., Xu Z., Xia T., Li M. (2016). Application of Remote Sensing-based Two-source Energy Balance Model for Mapping Field Surface Fluxes with Composite and Component Surface Temperatures. Agric. For. Meteorol..

[94] Guzinski R., Anderson M.C., Kustas W.P., Nieto H., Sandholt I. (2013). Using a Thermal-based Two Source Energy Balance Model with Time-differencing to Estimate Surface Energy Fluxes with Day-night MODIS Observations. Hydrol. Earth Syst. Sci..

[95] Santanello J.A., Friedl M.A. (2003). Diurnal Covariation in Soil Heat Flux and Net Radiation. J. Appl. Meteorol..

[96] Guzinski R., Nieto H., Jensen R., Mendiguren G. (2014). Remotely Sensed Land-surface Energy Fluxes at Sub-field Scale in Heterogeneous Agricultural Landscape and Coniferous Plantation. Biogeosciences.

[97] Guzinski R., Nieto H., Stisen S., Fensholt R. (2015). Inter-comparison of Energy Balance and Hydrological Models for Land Surface Energy Flux Estimation over a Whole River Catchment. Hydrol. Earth Syst. Sci..

[98] Bastiaanssen W.G.M. (1995). Regionalization of Surface Flux Densities and Moisture Indicators in Composite Terrain: A Remote Sensing Approach under Clear Skies in Mediterranean Climates.

[99] Bastiaanssen W.G. (2000). SEBAL-based Sensible and Latent Heat Fluxes in the Irrigated Gediz Basin, Turkey. J. Hydrol..

[100] Bastiaanssen W.G., Ahmad M.U.D., Chemin Y. (2002). Satellite Surveillance of Evaporative Depletion across the Indus Basin. Water Resour. Res..

[101] Ruhoff A.L., Paz A.R., Collischonn W., Aragao L.E., Rocha H.R., Malhi Y.S. (2012). A MODIS-based Energy Balance to Estimate Evapotranspiration for Clear-sky Days in Brazilian Tropical Savannas. Remote Sens..

[102] Sun Z., Wei B., Su W., Shen W., Wang C., You D., Liu Z. (2011). Evapotranspiration Estimation Based on the SEBAL Model in the Nansi Lake Wetland of China. Math. Comput. Model..

[103] Singh R., Senay G. (2016). Comparison of Four Different Energy Balance Models for Estimating Evapotranspiration in the Midwestern United States. Water.

[104] Bastiaanssen W., Noordman E., Pelgrum H., Davids G., Thoreson B., Allen R. (2005). SEBAL Model with Remotely Sensed Data to Improve Water-resources Management under Actual Field Conditions. J. Irrig. Drain. Eng..

[105] Gowda P.H., Chavez J.L., Colaizzi P.D., Evett S.R., Howell T.A., Tolk J.A. (2008). ET Mapping for Agricultural Water Management: Present Status and Challenges. Irrig. Sci..

[106] Jacob F., Olioso A., Gu X.F., Su Z., Seguin B. (2002). Mapping Surface Fluxes Using Airborne Visible, Near Infrared, Thermal Infrared Remote Sensing Data and a Spatialized Surface Energy Balance Model. Agronomie.

[107] Tasumi M., Allen R.G., Trezza R., Wright J.L. (2005). Satellite-based Energy Balance to Assess Within-population Variance of Crop Coefficient Curves. J. Irrig. Drain. Eng..

[108] Hunt E.R., Cavigelli M., Daughtry C.S., Mcmurtrey J.E., Walthall C.L. (2005). Evaluation of Digital Photography from Model Aircraft for Remote Sensing of Crop Biomass and Nitrogen Status. Precis. Agric..

[109] Gentine P., Entekhabi D., Heusinkveld B. (2012). Systematic Errors in Ground Heat Flux Estimation and Their Correction. Water Resour. Res..

[110] Li F., Kustas W.P., Prueger J.H., Neale C.M., Jackson T.J. (2005). Utility of Remote Sensing-based Two-source Energy Balance Model under Low-and High-vegetation Cover Conditions. J. Hydrometeorol..

[111] Feng J., Wang Z. (2013). A Satellite-based Energy Balance Algorithm with Reference Dry and Wet Limits. Int. J. Remote Sens..

[112] Long D., Singh V.P. (2013). Assessing the Impact of End-member Selection on the Accuracy of Satellite-based Spatial Variability Models for Actual Evapotranspiration Estimation. Water Resour. Res..

[113] Duggin M., Robinove C. (1990). Assumptions Implicit in Remote Sensing Data Acquisition and Analysis. Remote Sens..

[114] Lillesand T., Kiefer R.W., Chipman J. (2015). Remote Sensing and Image Interpretation.

[115] Wang J., Sammis T., Gutschick V., Gebremichael M., Miller D. (2009). Sensitivity Analysis of the Surface Energy Balance Algorithm for Land (SEBAL). Trans. ASABE.

[116] Allen R., Tasumi M., Trezza R., Kjaersgaard J. (2008). METRICTM–Mapping Evapotranspiration at High Resolution–Applications Manual for Landsat Satellite Imagery (Version 2.0.4).

[117] Laliberte A.S., Rango A., Herrick J. Unmanned aerial vehicles for rangeland mapping and monitoring: A comparison of two systems. Proceedings of the ASPRS Annual Conference.

[118] Hardin P.J., Hardin T.J. (2010). Small-scale Remotely Piloted Vehicles in Environmental Research. Geogr. Compass.

[119] Hardin P.J., Jensen R.R. (2011). Small-scale Unmanned Aerial Vehicles in Environmental Remote Sensing: Challenges and Opportunities. GISci. Remote Sens..

[120] Stark B., Chen Y. A framework of optimal remote sensing using small unmanned aircraft systems. Proceedings of the 12th IEEE/ASME International Conference on Mechatronic and Embedded Systems and Applications (MESA).

[121] Harwin S., Lucieer A. (2012). Assessing the Accuracy of Georeferenced Point Clouds Produced via Multi-view Stereopsis from Unmanned Aerial Vehicle (UAV) Imagery. Remote Sens..

[122] Lucieer A., Malenovskỳ Z., Veness T., Wallace L. (2014). HyperUAS—Imaging Spectroscopy from a Multirotor Unmanned Aircraft System. J. Field Robot..

[123] Turner D., Lucieer A., Watson C. (2012). An Automated Technique for Generating Georectified Mosaics from Ultra-high Resolution Unmanned Aerial Vehicle (UAV) Imagery, Based on Structure from Motion (SfM) Point Clouds. Remote Sens..

[124] Wallace L., Lucieer A., Watson C., Turner D. (2012). Development of a UAV-LiDAR System with Application to Forest Inventory. Remote Sens..

[125] Zhao T., Stark B., Chen Y., Ray A.L., Doll D. A detailed field study of direct correlations between ground truth crop water stress and normalized difference vegetation index (NDVI) from small unmanned aerial system (sUAS). Proceedings of the 2015 International Conference on Unmanned Aircraft Systems (ICUAS).

[126] Smith G.M., Milton E.J. (1999). The Use of the Empirical Line Method to Calibrate Remotely Sensed Data to Reflectance. Int. J. Remote Sens..

[127] Lelong C., Burger P., Jubelin G., Roux B., Labbé S., Baret F. (2008). Assessment of Unmanned Aerial Vehicles Imagery for Quantitative Monitoring of Wheat Crop in Small Plots. Sensors.

[128] Xiang H., Tian L. (2011). Method for Automatic Georeferencing Aerial Remote Sensing (RS) Images from an Unmanned Aerial Vehicle (UAV) Platform. Biosyst. Eng..

[129] Stark B., Zhao T., Chen Y. An analysis of the effect of the bidirectional reflectance distribution function on remote sensing imagery accuracy from small unmanned aircraft systems. Proceedings of the 2016 International Conference on Unmanned Aircraft Systems (ICUAS).

[130] Berni J.A., Zarco-Tejada P.J., Suárez L., Fereres E. (2009). Thermal and Narrowband Multispectral Remote Sensing for Vegetation Monitoring from an Unmanned Aerial Vehicle. IEEE Trans. Geosci. Remote Sens..

[131] Brown K., Rosenberg N.J. (1973). A Resistance Model to Predict Evapotranspiration and Its Application to a Sugar Beet Field 1. Agron. J..

[132] Sheng H., Chao H., Coopmans C., Han J., McKee M., Chen Y. Low-cost UAV-based thermal infrared remote sensing: Platform, calibration and applications. Proceedings of the 2010 IEEE/ASME International Conference on Mechatronics and Embedded Systems and Applications (MESA).

[133] Gade R., Moeslund T.B. (2014). Thermal Cameras and Applications: A Survey. Mach. Vis. Appl..

[134] Jones H.G., Serraj R., Loveys B.R., Xiong L., Wheaton A., Price A.H. (2009). Thermal Infrared Imaging of Crop Canopies for the Remote Diagnosis and Quantification of Plant Responses to Water Stress in the Field. Funct. Plant Biol..

[135] Gowen A., Tiwari B., Cullen P., McDonnell K., O’Donnell C. (2010). Applications of Thermal Imaging in Food Quality and Safety Assessment. Trends Food Sci. Technol..

[136] Martinelli F., Scalenghe R., Davino S., Panno S., Scuderi G., Ruisi P., Villa P., Stroppiana D., Boschetti M., Goulart L.R. (2015). Advanced Methods of Plant Disease Detection. A Review. Agron. Sustain. Dev..

[137] Fisher J.B., Melton F., Middleton E., Hain C., Anderson M., Allen R., McCabe M.F., Hook S., Baldocchi D., Townsend P.A. (2017). The Future of Evapotranspiration: Global Requirements for Ecosystem Functioning, Carbon and Climate Feedbacks, Agricultural Management, and Water Resources. Water Resour. Res..

[138] Niu H., Wang D., Chen Y. Estimating crop coefficients using linear and deep stochastic configuration networks models and UAV-based Normalized Difference Vegetation Index (NDVI). Proceedings of the 2020 International Conference on Unmanned Aircraft Systems (ICUAS).

[139] Niu H., Wang D., Chen Y. Estimating actual crop evapotranspiration using deep stochastic configuration networks model and UAV-based crop coefficients in a pomegranate orchard. Proceedings of the Autonomous Air and Ground Sensing Systems for Agricultural Optimization and Phenotyping V.

